# Short-term outcomes of centralization on surgical care for patients with anorectal malformations: retrospective cohort study

**DOI:** 10.1093/bjsopen/zraf155

**Published:** 2026-01-20

**Authors:** Malin af Petersens, Pernilla Stenström, Helena Borg, Johan Danielson, Lisa Örtqvist, Anna Gunnarsdottir, Jenny Oddsberg, Elisabet Gustafson, Christina Graneli, Kristine Hagelsteen, Louise Tofft, Tomas Wester

**Affiliations:** Department of Women's and Children's Health, Karolinska Institutet, Stockholm, Sweden; Department of Otorhinolaryngology, Karolinska University Hospital, Stockholm, Sweden; Department of Clinical Sciences Lund, Pediatrics, Lund University, Lund, Sweden; Department of Pediatric Surgery, Skåne University Hospital, Lund, Sweden; Department of Pediatric Surgery, Drottning Silvia's Children's Hospital, Göteborg, Sweden; Department of Women´s and Children's Health, Uppsala University, Uppsala, Sweden; Department of Women's and Children's Health, Karolinska Institutet, Stockholm, Sweden; Department of Pediatric Surgery, Karolinska University Hospital, Stockholm, Sweden; Department of Women's and Children's Health, Karolinska Institutet, Stockholm, Sweden; Department of Pediatric Surgery, Karolinska University Hospital, Stockholm, Sweden; Department of Women's and Children's Health, Karolinska Institutet, Stockholm, Sweden; Department of Pediatric Surgery, Karolinska University Hospital, Stockholm, Sweden; Department of Women´s and Children's Health, Uppsala University, Uppsala, Sweden; Department of Clinical Sciences Lund, Pediatrics, Lund University, Lund, Sweden; Department of Pediatric Surgery, Skåne University Hospital, Lund, Sweden; Department of Clinical Sciences Lund, Pediatrics, Lund University, Lund, Sweden; Department of Pediatric Surgery, Skåne University Hospital, Lund, Sweden; Department of Clinical Sciences Lund, Pediatrics, Lund University, Lund, Sweden; Department of Pediatric Surgery, Skåne University Hospital, Lund, Sweden; Department of Women's and Children's Health, Karolinska Institutet, Stockholm, Sweden; Department of Pediatric Surgery, Karolinska University Hospital, Stockholm, Sweden

**Keywords:** paediatric, surgery

## Abstract

**Background:**

The Swedish National Board of Health and Welfare centralized the surgical care of patients with anorectal malformations from four to two centres in 2018. This retrospective review compares short-term complications after anorectal reconstruction before and after centralization.

**Methods:**

Hospital records of all infants in Sweden who underwent reconstruction of an anorectal malformation between 1 July 2013 and 30 June 2023 were reviewed and divided in two 5-year periods: before and after centralization. The main outcomes were unplanned readmissions and surgical procedures requiring general anaesthesia up to 90 days after reconstruction, as well as early complications classified according to the Clavien–Madadi system up to 30 days after the procedure.

**Results:**

Before centralization, 173 infants underwent anorectal reconstruction, compared with 176 infants after centralization. Patient groups were comparable with respect to associated malformations and type of anorectal malformation. Before centralization, 80 infants (46.2%) had a colostomy before the anorectal reconstruction, compared with 89 infants (50.6%) after centralization (*P* = 0.454). Anorectal reconstruction was performed at a median age of 61 and 47 days of age before and after centralization, respectively (*P* = 0.794). Unplanned readmissions up to 90 days after anorectal reconstruction were needed in 12 infants (6.9%) before centralization, compared with 22 infants (12.5%) after centralization (*P* = 0.104). Unplanned surgical procedures under general anaesthesia were required in 20 (11.6%) and 22 (12.5%) infants before and after centralization, respectively (*P* = 0.870). Complications (Clavien–Madadi grade III–V) within 30 days after anorectal reconstruction were seen in 16 (9.2%) and 12 (6.8%) infants before and after centralization, respectively (*P* = 0.436).

**Conclusion:**

Centralization of the surgical care of patients with anorectal malformations in Sweden did not seem to have an impact on short-term complications.

## Teaser

This retrospective study evaluated the impact of centralizing surgical care for patients with anorectal malformations (ARM) in Sweden. Short-term postoperative outcomes before and after centralization were compared, with a focus on readmissions and unplanned surgical procedures within 90 days, as well as Clavien–Madadi grade III–IV complications within 30 days. The findings suggest that centralization did not significantly affect these short-term outcomes.

## Introduction

Anorectal malformations (ARM) comprise a wide spectrum of congenital anomalies, with a birth prevalence in Sweden of approximately 1 in 3000^1^. Defects range from mild anterior displacement of the anus to complex malformations, involving both the hindgut and urogenital tract. Associated anomalies are encountered in 50–60% of individuals with ARM^1^ and contribute significantly to morbidity.

Surgical techniques to treat ARM have progressively developed over the past two centuries. The current surgical technique for ARM repair was introduced in the 1980s by Peña and de Vries when they performed the first posterior sagittal anorectoplasty (PSARP)^2^, which compares favourably with previous techniques^2,3^ and has become the standard of care for the repair of ARM. Traditionally, the repair includes a three-stage approach: a primary colostomy, anorectal reconstruction, and closure of the colostomy. However, single-stage PSARP has increasingly been performed in less complex malformations^4^. In recent years, laparoscopy-assisted repair has become an alternative approach^5,6^.

Several complications have been associated with the surgical repair of ARM. These include wound infections, wound dehiscence, prolapse, anal strictures, and urinary tract injuries^7–9^. To grade the severity of postoperative complications, the Clavien–Madadi classification was recently developed^10^. The Clavien–Madadi classification is a modification of the Clavien–Dindo classification that better fits the paediatric population and has undergone validation for clinical and scientific applications^11^.

The Swedish National Board of Health and Welfare decided in 2017 that the highly specialized care of ARM should be centralized to Region Stockholm (Karolinska University Hospital) and Region Skåne (Skåne University Hospital) from 1 July 2018. Before this, four tertiary paediatric surgical centres provided treatment for patients with ARM in Sweden. The impact of this centralization on patient outcomes and quality of care remains uncertain.

The concentration of complex surgical care in adult surgery to specialized centres has been shown to positively influence the quality of care^12^. The beneficial effect of centralization seen in adult surgery is particularly evident in procedures such as pancreatic and oesophageal surgery, where data indicate^12^ that centralization has been associated with reduced postoperative mortality and morbidity. Another positive impact of centralization has been reduced failure-to-rescue rates in patients with complications^12^. Moreover, readmission to the index hospital has been associated with a lower risk of mortality compared with readmission to non-specialized hospitals^12^.

Although evidence regarding the impact of centralization in paediatric surgery is limited, studies indicate that centralization of portoenterostomy procedures for biliary atresia improves outcomes^13–16^. Recent evidence also suggests that outcomes of the surgical management of neuroblastomas^17^, as well as paediatric liver tumours^18^, may improve following centralization. Data are very limited regarding the impact of the centralization of surgical procedures for ARM^19^.

The aim of this study was to assess short-term outcomes after anorectal reconstruction before and after the centralization of the management of ARM in Sweden.

## Methods

### Study design

This was a retrospective observational study. The study was reported in accordance with the STROBE guidelines for observational studies (*supplementary methods*).

### Setting

**Fig. 1:**
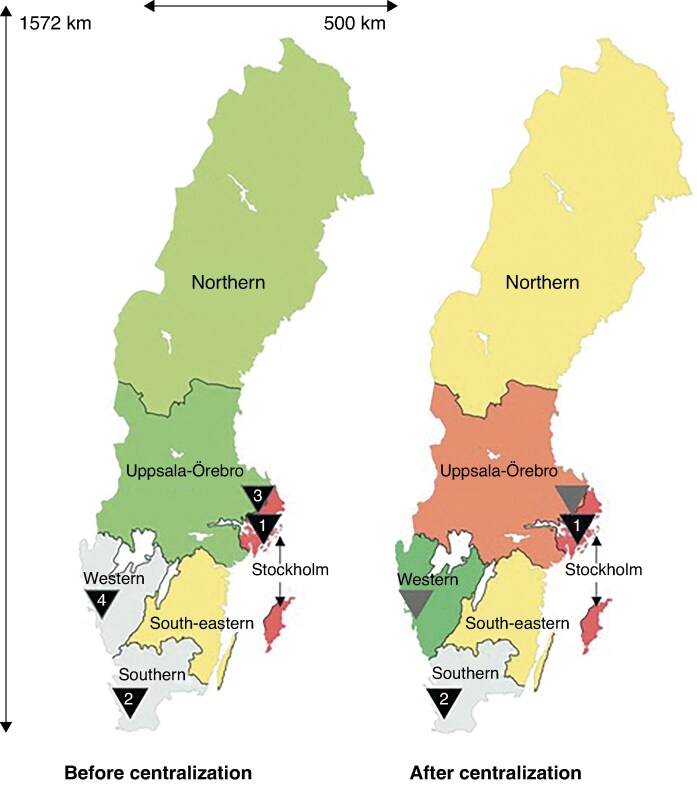
Maps of Sweden showing locations of the tertiary paediatric surgery centres before and after centralization Maps show the location of the four tertiary paediatric surgery centres before centralization (left) and the two centres after centralization (right). Black arrowheads indicate active centres; grey arrowheads indicate previous treating centres. 1, Karolinska University Hospital, Stockholm; 2, Skåne University Hospital, Lund; 3, University Children’s Hospital, Uppsala; 4, Queen Silvia Children's Hospital, Gothenburg. Reproduced and adapted from Söderström *et al.*^20^ Licensed under Creative Commons licence CC BY.

The study was conducted nationwide from 1 July 2013 to 30 June 2023, with data retrieved from the four tertiary paediatric surgery centres in Sweden (*Fig. 1*). These centres are the Department of Paediatric Surgery at Skåne University Hospital in Lund, Karolinska University Hospital in Stockholm, University Children’s Hospital in Uppsala, and Queen Silvia Children’s Hospital in Gothenburg. All children who underwent anorectal reconstruction of an ARM during the specified timeframe were operated on in one of these four centres. After 1 July 2018, the surgical care of patients with ARM was centralized to Karolinska University Hospital and Skåne University Hospital.

### Participants

All children who underwent anorectal reconstruction of ARM at one of the Swedish tertiary paediatric surgery centres between 1 July 2013 and 30 June 2023 were included in the study. Eligible patients were identified in the participating hospital’s electronic medical record systems. Patients reconstructed for any type of ARM, classified according to Krickenbeck^21^, were included.

Patients diagnosed with ARM who did not receive surgical intervention or only required anal dilatation were excluded from the study. In addition, patients who underwent secondary anorectal reconstruction at one of the four tertiary paediatric surgery centres in Sweden after primary reconstruction in another country were excluded.

The specified time period was split into two consecutive 5-year segments: before and after centralization. Patients were then categorized into two groups. The first group included patients who underwent anorectal reconstruction before centralization, and the second group comprised those who underwent the reconstruction after centralization.

### Data sources and variables

All data were collected retrospectively from the electronic medical records of each hospital. Data collection was performed by one designated researcher at each participating hospital. Data were pseudonymized and subsequently consolidated into a Microsoft^®^ Excel (Microsoft, Redmond, WA, USA) spreadsheet.

### Patient characteristics

Demographic data, including sex, gestational age, and birthweight, and the specific centre where the repair was conducted were collected from the medical records. In addition, data were retrieved regarding the presence of a primary stoma, the age at which the stoma was established, the age at anorectal reconstruction, and the presence of associated malformations or syndromes.

Furthermore, the type of ARM and anorectal reconstruction of each patient was recorded. The ARM type was categorized according to the Krickenbeck classification as perineal fistulas, rectourethral fistulas (bulbar and prostatic), rectovesical fistulas, vestibular fistulas, cloacal malformations, patients with no fistula, anal stenosis, and rare variants^21^. The type of anorectal reconstruction was categorized into PSARP, PSARP with laparoscopy, PSARP with laparotomy, limited PSARP, posterior sagittal anorectal vaginal urethral plasty (PSARVUP), and other.

### Surgical outcomes

The outcomes included the length of hospital stay after the anorectal reconstruction, unplanned surgical procedures requiring general anaesthesia within 90 days of the reconstruction, unplanned readmission within 90 days of the reconstruction, and postoperative complications within 30 days of the reconstruction.

Unplanned readmissions and surgical procedures encompassed events related to the anorectal reconstruction. These further comprised events occurring not only at the index hospital but also at other hospitals in Sweden. The length of hospital stay accounted for both the initial admission at the index hospital and any subsequent transfers to other hospitals in Sweden. The data from medical records kept by other hospitals were retrieved through the National Patient Summary, providing direct access to medical records from all healthcare providers operated by the 21 regions in Sweden.

Postoperative complications were graded using the Clavien–Madadi classification^10^. Complications classified as IIIA, IIIB, IV, and V were included for analysis. Each patient was categorized as either having experienced one or more of these complications or not. In addition, the number of occurrences of each complication type was analysed. Each patient was categorized by the highest degree of complication they experienced. If a patient had multiple complications, only the highest grade was recorded.

### Bias

Inclusion and exclusion criteria were clearly defined to control for potential selection bias. To enhance the process of data extraction from medical records, data collection was conducted separately at each hospital. This approach may reduce information bias by facilitating data extraction. To further reduce information bias, complications graded as I and II were excluded because these were considered more difficult to identify retrospectively in the medical records.

To further ensure consistency in data collection and assessment, the refined and validated grading instrument for unexpected events in paediatric surgery, namely the Clavien–Madadi classification, was used to grade complications^11^.

### Statistical analysis

Categorical variables are presented as frequencies and proportions. Continuous variables are presented as the median and interquartile range (i.q.r.) or as the mean and standard deviation (s.d.), depending on the normality of data distribution. The two groups, namely before and after centralization, were compared using Fisher’s exact test or the χ^2^ test for categorical variables and the Mann–Whitney *U* test for continuous variables. *P* < 0.05 was considered statistically significant, all tests were two-sided.

### Ethical considerations

This study was approved by the Swedish Ethical Review Authority (2023-02650-01).

## Results

### Participants

In all, 349 patients were included in the study. There were 173 patients in the group that underwent anorectal reconstruction before centralization and 176 patients in the group that underwent reconstruction after centralization.

### Descriptive data

The two groups were comparable regarding sex, gestational age, birthweight, and the presence of associated malformations or syndromes (*Table 1*). There were no statistically significant differences between the two groups in terms of the need for a primary stoma (*P* = 0.454) or the age at which the primary stoma was established (*P* = 0.300; *Table 1*).

**Table 1:** Patient characteristics before and after the centralization of surgical care of patients with anorectal malformations

	Before centralization	After centralization	P*	Missing data
**Hospital**				
Stockholm	50 (28.9%)	101 (57.4%)		
Lund	30 (17.3%)	74 (42.0%)		
Gothenburg	39 (22.5%)	1 (0.6%)		
Uppsala	54 (31.2%)	0 (0.0%)		
Sex ratio (male : female)	1 : 0.84	1 : 0.64	0.235	
Gestational age (weeks), mean(s.d.)	38.18(2.55)	38.29(2.57)	0.693†	15 (4.3%)
Birthweight (g), mean(s.d.)	3127.95(701.54)	3255.92(728.55)	0.105†	19 (5.4%)
Associated malformations or syndrome	101 (58.4%)	112 (63.6%)	0.325	
**Type of anorectal malformation**			NA	
Perineal fistula	88 (50.9%)	81 (46.0%)		
Rectourethral fistula	24 (13.9%)	34 (19.3%)		
Rectovesical fistula	3 (1.7%)	7 (4.0%)		
Vestibular fistula	24 (13.9%)	34 (19.3%)		
Cloaca	7 (4.0%)	7 (4.0%)		
No fistula	24 (13.9%)	7 (4.0%)		
Anal stenosis	1 (0.6%)	1 (0.6%)		
Primary stoma	80 (46.2%)	89 (50.6%)	0.454	
Age at primary stoma (days), median (i.q.r.)	1.00 (1.00–2.00)	1.00 (1.00–2.00)	0.300†	
Age at anorectal reconstruction (days), median (i.q.r.)	61.00 (3.00–138.00)	47.00 (4.00–134.25)	0.794†	

Values are *n* (%) unless otherwise stated. s.d., standard deviation; NA, not available; i.q.r., interquartile range. *Fisher's exact test, except †Mann–Whitney *U* test.

### Outcome data and main results

The main surgical outcome parameters showed no significant differences before and after centralization. These parameters include length of hospital stay after anorectal reconstruction, unplanned surgical procedures, readmissions within 90 days of reconstruction, and the occurrence of a complication within 30 days of reconstruction.

The median length of hospital stay after anorectal reconstruction was 7 days in both groups (*P* = 0.680; *Table 2*). There were more patients requiring readmission within 90 days after the anorectal reconstruction after centralization, but the difference was not statistically significant (*P* = 0.104; *Table 2*). Before centralization, 20 patients (11.6%) underwent an unplanned surgical procedure requiring general anaesthesia, compared with 22 patients (12.5%) after centralization (*P* = 0.870). Clavien–Madadi grade III–V complications were observed in more patients before centralization, but the difference was not statistically significant (*P* = 0.436; *Table 2*). However, the distribution of grade IIIA and IIIB complications differed significantly, with grade IIIB complications more frequent before centralization and grade IIIA complications more frequent after centralization (*P* = 0.018; *Table 2*).

**Table 2:** Postoperative outcomes before and after the centralization of surgical care of patients with anorectal malformations

	Before centralization	After centralization	P*
Total LOS after anorectal reconstruction (days), median (i.q.r.)	7.00 (5.00–9.00)	7.00 (3.00–10.00)	0.680†
Unplanned readmission within 90 days	12 (6.9%)	22 (12.5%)	0.104
Unplanned surgical procedure within 90 days	20 (11.6%)	22 (12.5%)	0.870
**Complications after anorectal reconstruction‡**	16 (9.2%)	12 (6.8%)	0.436
Grade IIIA complications	6 (38%)	9 (75%)	
Grade IIIB complications	9 (56%)	1 (8%)	
Grade IV complications	1 (6%)	2 (17%)	
Grade V complications	0 (0%)	0 (0%)	

Values are *n* (%) unless otherwise stated. ‡Grade III–V complications according to the Clavien–Madadi classification. LOS, length of hospital stay; i.q.r., interquartile range. *Fisher's exact test, except †Mann–Whitney *U* test.

## Discussion

This retrospective observational study aimed to assess the impact of centralization on short-term complications after anorectal reconstruction in patients with ARM. The study did not show any significant differences in the observed outcomes after centralization.

Centralization has significantly improved outcomes of biliary atresia^13–16^ and demonstrated clear benefits in various other areas of paediatric surgery^17,18,22^. It has been discussed whether improved outcomes are more influenced by hospital volumes or surgeon volumes. Hospital factors such as intensive care unit availability and well-trained staff may add to the impact of hospital volume, particularly within the context of the failure-to-rescue concept^12^.

The lack of significant changes in short-term complications after centralization in this study may be interpreted in several ways. Before the centralization of ARM in 2018, care was already concentrated to four tertiary paediatric surgery centres in Sweden, with an average of 8.7 procedures per hospital per year. After centralization, the number of centres was reduced to two, resulting in an average of 17.6 procedures per hospital per year.

A study from Germany^19^ reported 2060 anorectal reconstructions of ARM between 2016 and 2021 across 113 hospitals, averaging 3 surgeries per hospital per year. That study also showed that major early complications occurred in 24.5% of these reconstructive procedures^19^. The European Reference Network for rare urogenital diseases and complex conditions (eUROGEN) recommends a minimum caseload of ten anorectal reconstructions per year to improve the quality of care for ARM patients^23^. One can speculate that countries with a large number of centres, with limited caseload, would experience more pronounced effects of centralization. The impact of centralization is likely related to the healthcare infrastructure where it is implemented.

In Finland, where there is a geographically uneven distribution of paediatric surgical patients and limited caseload, a different approach has been adopted. Paediatric surgeons established the Finnish Pediatric Surgery Hub (FPSH), enabling national collaboration and sharing of expertise. FPSH coordinates patient management through Helsinki University Hospital, selecting the place of care and surgical team based on case complexity, offering an alternative to classic centralization^24^.

Similarly, centralization can be challenging in Sweden with its unevenly distributed population of approximately 10.5 million and a low population density of 26 /km^2^. The country spans over 1500 km from north to south and travelling from the northernmost areas to Stockholm can take up to 15 hours by car. Large areas with low population density rely on well functioning transport facilities to connect remote regions. Centralization in these areas can lead to longer travel times and logistical issues for families. However, findings from earlier studies suggest that the most important factor from a patient perspective in decisions regarding centralization is the quality of care rather than travel distances^21,25^.

In addition, several patient organizations support the minimal case number recommendation recommended by eUROGEN, and advocate for centralization to enhance surgical outcomes, overall treatment quality, and long-term quality of life^26^.

Long distances to the tertiary medical facility could further increase the risk of misdiagnosis or delayed diagnosis, particularly for rare diseases^27^. Nevertheless, the results of the present study suggest that centralization did not adversely affect patient outcomes. Moreover, the median age at primary stoma and anorectal reconstruction did not differ significantly between the two groups.

This study is among the first to evaluate the impact of the centralization of surgical care for ARM. The generalizability of the findings is strengthened by the nationwide coverage of the study, which included a diverse patient population across multiple centres. The prevalence of ARM and sex ratio observed in this study align with previous reports^1,28^.

However, because the study was conducted within a single country, the results may still be influenced by factors specific to the healthcare system, travel distances, and management of ARM in Sweden, which could affect external validity.

Several limitations should be considered when interpreting the results of this study. Although this was a nationwide study conducted over a 10-year period, the relatively limited number of patients may still affect the statistical power of the study, increasing the risk of a type II error. In addition, the retrospective design of the study may introduce information bias, because it does not ensure the accuracy and completeness of all collected variables. Furthermore, the study design could lead to selection bias due to differences between the populations before and after centralization. However, no statistically significant differences were observed between the groups in terms of demographic or descriptive perioperative data.

A washout period after centralization could have been valuable to allow time for adaptation across the involved hospitals. However, due to the limited number of patients, the full 5-year period was included to ensure a sufficiently large cohort for analysis.

## Supplementary Material

zraf155_Supplementary_Data

## Data Availability

Raw data will be made available upon request.
