# Supplementary material for: Short-term outcomes of centralization on surgical care for patients with anorectal malformations: retrospective cohort study
Source: BJS Open. 2026 Jan 20;10(1):zraf155. doi: 10.1093/bjsopen/zraf155 (PMC12817992; doi:10.1093/bjsopen/zraf155)
Supplement: zraf155_Supplementary_Data [file zraf155_supplementary_data.docx]

**Short-Term outcomes of Centralization on Surgical Care for Patients with Anorectal malformations: Retrospective cohort study**

Malin af Petersens^1,2^, Pernilla Stenström^3,4^, Helena Borg^5^, Johan Danielson^6^, Lisa Örtqvist^1,7^, Anna Gunnarsdottir^1,7^, Jenny Oddsberg^1,7^, Elisabet Gustafson^6^, Christina Graneli^3,4^, Kristine Hagelsteen^3,4^, Louise Tofft^3,4^, Tomas Wester^1,7^
^1^ Department of Women's and Children's Health, Karolinska Institutet, Stockholm, Sweden
^2^ Department of Otorhinolaryngology, Karolinska University Hospital, Stockholm, Sweden
^3^ Department of Clinical Sciences Lund, Pediatrics, Lund university, Lund, Sweden

^4^ Department of Pediatric Surgery, Skåne University Hospital, Lund, Sweden
^5^ Department of Pediatric Surgery, Drottning Silvia's Children's Hospital, Göteborg, Sweden
^6^ Department of Women´s and Children's health, Uppsala University, Uppsala, Sweden
^7^ Department of Pediatric Surgery, Karolinska University Hospital, Stockholm, Sweden

**Corresponding author.** Malin af Petersens, Tomtebodavägen 18A, 171 77 Solna, [malin.af.petersens@ki.se](mailto:malin.af.petersens@ki.se).

**Supplementary Materials - Index**

| **Supplementary Methods** |  |
| --- | --- |
| STROBE checklist | *page 2-4* |
|  |  |

**Supplementary Methods**

STROBE Statement—checklist of items that should be included in reports of observational studies

|  | Item No. | Recommendation | Page  No. | Relevant text from manuscript |
| --- | --- | --- | --- | --- |
| **Title and abstract** | 1 | (*a*) Indicate the study’s design with a commonly used term in the title or the abstract | 1-2 | Retrospective cohort study |
|  |  | (*b*) Provide in the abstract an informative and balanced summary of what was done and what was found | 2 |  |
| Introduction | | | |  |
| Background/ rationale | 2 | Explain the scientific background and rationale for the investigation being reported | 2-3 |  |
| Objectives | 3 | State specific objectives, including any prespecified hypotheses | 3 |  |
| Methods | | | |  |
| Study design | 4 | Present key elements of study design early in the paper | 3 | This was a retrospective observational study. |
| Setting | 5 | Describe the setting, locations, and relevant dates, including periods of recruitment, exposure, follow-up, and data collection | 3 | The study was conducted nationwide from 1 July 2013 to 30 June 2023, with data retrieved from the four tertiary paediatric surgery centres in Sweden. |
| Participants | 6 | (*a*) *Cohort study*—Give the eligibility criteria, and the sources and methods of selection of participants. Describe methods of follow-up  *Case-control study*—Give the eligibility criteria, and the sources and methods of case ascertainment and control selection. Give the rationale for the choice of cases and controls  *Cross-sectional study*—Give the eligibility criteria, and the sources and methods of selection of participants | 4 | All children who underwent anorectal reconstruction of ARM at one of the Swedish tertiary paediatric surgery centres between 1 July 2013 and 30 June 2023 were included in the study. |
|  |  | (*b*) *Cohort study*—For matched studies, give matching criteria and number of exposed and unexposed  *Case-control study*—For matched studies, give matching criteria and the number of controls per case |  |  |
| Variables | 7 | Clearly define all outcomes, exposures, predictors, potential confounders, and effect modifiers. Give diagnostic criteria, if applicable | 4-5 | The outcomes included the length of hospital stay after the anorectal reconstruction, unplanned surgical procedures requiring general anaesthesia within 90 days of the reconstruction, unplanned readmission within 90 days of the reconstruction as well as post-operative complications within 30 days of the reconstruction. |
| Data sources/ measurement | 8* | For each variable of interest, give sources of data and details of methods of assessment (measurement). Describe comparability of assessment methods if there is more than one group | 4-5 | All data were collected retrospectively from the electronic medical records of each hospital, respectively. |
| Bias | 9 | Describe any efforts to address potential sources of bias | 5 |  |
| Study size | 10 | Explain how the study size was arrived at |  |  |

| Quantitative variables | 11 | Explain how quantitative variables were handled in the analyses. If applicable, describe which groupings were chosen and why | 5 |  |
| --- | --- | --- | --- | --- |
| Statistical methods | 12 | (*a*) Describe all statistical methods, including those used to control for confounding | 5 |  |
|  |  | (*b*) Describe any methods used to examine subgroups and interactions |  |  |
|  |  | (*c*) Explain how missing data were addressed |  |  |
|  |  | (*d*) *Cohort study*—If applicable, explain how loss to follow-up was addressed  *Case-control study*—If applicable, explain how matching of cases and controls was addressed  *Cross-sectional study*—If applicable, describe analytical methods taking account of sampling strategy |  |  |
|  |  | (*e*) Describe any sensitivity analyses |  |  |
| Results | | | | |
| Participants | 13* | (a) Report numbers of individuals at each stage of study—eg numbers potentially eligible, examined for eligibility, confirmed eligible, included in the study, completing follow-up, and analysed | 5 | A total of 349 patients were included in the study. There were 173 patients in the group that underwent anorectal reconstruction before centralization and 176 patients in the group that underwent reconstruction after centralization. |
|  |  | (b) Give reasons for non-participation at each stage |  |  |
|  |  | (c) Consider use of a flow diagram |  |  |
| Descriptive data | 14* | (a) Give characteristics of study participants (eg demographic, clinical, social) and information on exposures and potential confounders | 5-6 | The two groups were comparable regarding sex, gestational age, birth weight and the presence of associated malformations or syndromes (Table 1). |
|  |  | (b) Indicate number of participants with missing data for each variable of interest |  |  |
|  |  | (c) *Cohort study*—Summarise follow-up time (eg, average and total amount) |  |  |
| Outcome data | 15* | *Cohort study*—Report numbers of outcome events or summary measures over time | 6 | The main surgical outcome parameters showed no significant differences before and after centralization. |
|  |  | *Case-control study—*Report numbers in each exposure category, or summary measures of exposure |  |  |
|  |  | *Cross-sectional study—*Report numbers of outcome events or summary measures |  |  |
| Main results | 16 | (*a*) Give unadjusted estimates and, if applicable, confounder-adjusted estimates and their precision (eg, 95% confidence interval). Make clear which confounders were adjusted for and why they were included | 6 |  |
|  |  | (*b*) Report category boundaries when continuous variables were categorized |  |  |
|  |  | (*c*) If relevant, consider translating estimates of relative risk into absolute risk for a meaningful time period |  |  |

| Other analyses | 17 | Report other analyses done—eg analyses of subgroups and interactions, and sensitivity analyses |  |  |
| --- | --- | --- | --- | --- |
| Discussion | | | | |
| Key results | 18 | Summarise key results with reference to study objectives | 6 |  |
| Limitations | 19 | Discuss limitations of the study, taking into account sources of potential bias or imprecision. Discuss both direction and magnitude of any potential bias | 7 |  |
| Interpretation | 20 | Give a cautious overall interpretation of results considering objectives, limitations, multiplicity of analyses, results from similar studies, and other relevant evidence | 6-7 |  |
| Generalisability | 21 | Discuss the generalisability (external validity) of the study results | 7 |  |
| Other information | |  | | |
| Funding | 22 | Give the source of funding and the role of the funders for the present study and, if applicable, for the original study on which the present article is based | 1 |  |
